# Triple-Negative Breast Cancer: Molecular Subtypes; Immune Escape; Limitations of Current Immunotherapy; and the BTLA/HVEM/CD160 Axis as an Emerging Target

**DOI:** 10.3390/cimb48050535

**Published:** 2026-05-20

**Authors:** Bernardo L. Rapoport, Ronald Anderson, Daniel van Tonder, Teresa Smit, Theresa M. Rossouw, Carol-Ann Benn, Helen C. Steel

**Affiliations:** 1 The Clinical and Translational Research Unit, Medical Oncology Centre of Rosebank, Saxonwold, Johannesburg 2196, South Africa; ronald.anderson@rosebankoncology-ctru.co.za (R.A.); danielvantonder@rosebankoncology-ctru.co.za (D.v.T.); tsmit@rosebankoncology-ctru.co.za (T.S.); 2 Centre for Innovative Immunology Research and Education, Department of Paediatrics and Child Health, Faculty of Health Sciences, University of Pretoria, Prinshof, Pretoria 0083, South Africa; theresa.rossouw@up.ac.za (T.M.R.); helen.steel@up.ac.za (H.C.S.); 3 Department of Immunology, Faculty of Health Sciences, University of Pretoria, Prinshof, Pretoria 0083, South Africa; breastcare@bcce.co.za; 4 Breast Care Centre of Excellence, Netcare Milpark Hospital, Parktown, Johannesburg 2193, South Africa

**Keywords:** BTLA, CD160, HVEM, immune checkpoint proteins, immune escape, immunotherapy, PD-1, PD-L1, triple-negative breast cancer

## Abstract

Triple-negative breast cancer is an aggressive and heterogeneous type of invasive breast cancer (BC) in which the cancer cells lack estrogen and progesterone receptors, as well as expression of the human epidermal growth factor 2 protein. This cancer tends to grow and spread faster than other BC subtypes, and is associated with a poor prognosis due to early visceral and neurological recurrences. Multidisciplinary management includes surgery, chemotherapy, radiation therapy, and immunotherapy with targeted immune checkpoint inhibitors (ICIs). The introduction of ICIs has improved outcomes in patients with TNBC, particularly in the metastatic and neoadjuvant settings. Despite these advances, a significant proportion of patients either do not respond to treatment or develop resistance to it. TNBC mortality remains high, underscoring the urgent need to identify novel prognostic and predictive biomarkers to overcome resistance to immunotherapy. Following a brief overview of the clinical features and established biomarkers of TNBC, the current review focuses on immune checkpoint proteins (ICPs) beyond PD-1 and PD-L1, and on the potential use of soluble ICPs rather than the well-established membrane-bound assays. These soluble ICPs are produced through the alternative splicing of messenger (m)RNA or the cleavage/shedding of membrane-bound proteins. This is followed by an overview of current treatment and novel predictive targets in TNBC. Additionally, the involvement of the B- and T-lymphocyte attenuator (BTLA)/herpes virus entry mediator (HVEM)/CD160 pathway and its role in the pathogenesis of BC and TNBC are reviewed, highlighting the potential use of BTLA and HVEM as biomarkers.

## 1. Introduction

Triple-negative breast cancer (TNBC) is an aggressive and heterogeneous type of invasive breast cancer (BC) in which the cancer cells lack estrogen receptors (ERs), progesterone receptors (PRs), and expression of the human epidermal growth factor 2 (HER2) protein [1]. TNBC tends to grow and spread faster than other BC subtypes, and has a higher risk of recurrence, especially within the first few years after treatment [1]. These early recurrences include visceral recurrences and are associated with a poor prognosis. TNBC is primarily managed with surgery, chemotherapy, radiation therapy, and immunotherapy with targeted immune checkpoint inhibitors (ICIs). Recent advances in the treatment of TNBC with inhibitors of programmed cell death protein 1 (PD-1) and its ligand (PD-L1) (pembrolizumab and atezolizumab, respectively)—including their approval as a standard of care when combined with chemotherapy—have resulted in improved outcomes for both early-stage and metastatic disease [2,3,4].

The introduction of ICIs has improved outcomes in patients with TNBC, particularly in the metastatic and neoadjuvant settings. Regardless of these developments, a significant proportion of patients either fail to respond or develop resistance after an initial response [5].

There is an unmet medical need for the identification of predictive biomarkers to improve the treatment of these patients. Following a consideration of the clinical characteristics and established biomarkers of TNBC, this review highlights immune checkpoint proteins (ICPs) beyond PD-1 and PD-L1, as well as the potential application of soluble ICPs in contrast to the conventional membrane-bound assays. Furthermore, the current treatment strategies and novel predictive targets in TNBC are discussed, exploring the potential use of the B- and T-lymphocyte attenuator (BTLA) and herpes virus entry mediator (HVEM) as biomarkers. The review presents a selective focus on TNBC, integrating updates in molecular biology, biochemistry, and immunotherapy for this lethal disease. The manuscript concludes with an overview of the roles of the BTLA/HVEM/CD160 pathway in the pathogenesis of BC and TNBC.

This manuscript is an extensive, non-systematic narrative literature review, comprising current and topical publications, as far as possible, based on searches in prominent databases, including, but not restricted to, PubMed, PMC, and Medline. All authors conducted independent searches to ensure maximum selection and limit selection bias.

## 2. Breast Cancer Subtypes

As mentioned above, there are four major biological types of BC that are defined based on gene expression profiles and surrogate immunohistochemistry (IHC) markers, including ER, PR, HER2, and antigen Kiel-67 (Ki-67) expression [6]. The luminal subtype [hormone receptor (HR)-positive] is defined as ER- and/or PR-positive, HER2-negative. This luminal subtype is further categorized into luminal A, which includes a high expression of ERs/PRs, HER2-negative or -low, and variable Ki-67 expression (<14–20%). Luminal A has the best prognosis and is generally responsive to endocrine therapy. Luminal B is defined as ER-positive/PR-low or -negative, HER2-negative or HER2-low, and Ki-67-high (≥20%). This subtype is more aggressive, often requiring treatment with chemotherapy and endocrine therapy with cyclin-dependent kinase 4 and 6 (CDK4/6) inhibitors [7]. The HER2-enriched subtype is categorized as HER2-overexpressed [IHC 3+ or fluorescence in situ hybridization (FISH)-amplified] and ER-/PR-negative. This subset is highly proliferative, aggressive, and has a poor prognosis without targeted therapy, such as trastuzumab, pertuzumab, trastuzumab deruxtecan, and others [7]. These subtypes of BC are biologically distinct from those of TNBC—which is defined as being ER-negative, PR-negative, and HER2-negative—and are discussed in more detail below [7,8].

## 3. Triple-Negative Breast Cancer

This form of BC represents between 15% and 20% of all BCs. It is often associated with younger age, higher-grade tumors, and the presence of breast cancer gene 1 (*BRCA1*) mutations. In addition to high Ki-67 expression, TNBC is associated with basal-like histology and, similar to the other BC subtypes described above, has an aggressive clinical course. Early recurrence is the hallmark of this subtype, which includes visceral recurrences and is associated with a poor prognosis. Additionally, most recurrences involve metastases to the lung, liver, and central nervous system and typically occur within the first three years [9]. Contrary to luminal A tumors, late recurrence after 5 years is uncommon. The presence of “bone-only” metastases is less common than in patients with HR-positive disease. Additionally, the recurrence risk declines speedily after 5 years; furthermore, patients who remain disease-free beyond this period have a relatively low ongoing risk [9,10].

### Triple-Negative Breast Cancer Subtypes

Lehmann and collaborators originally proposed six subtypes of TNBC: two basal-like subtypes (BL1 and BL2), a mesenchymal (M), a mesenchymal stem-like (MSL), an immunomodulatory (IM), and a luminal androgen receptor (LAR) subtype [11]. This classification was subsequently divided into four subtypes, BL, M-like, IM, and LAR, which are primarily used in the research setting but not routinely in clinical practice [12,13,14].

BL1 and BL2 are molecular subtypes of TNBC that display gene expression patterns similar to those of basal/myoepithelial cells in the normal breast. Approximately 70–80% of TNBCs are basal-like, as determined by gene expression profiling. The BL1 subtype is strongly enriched in cell cycle and DNA damage response (DDR) pathways, and is often linked to *BRCA1* dysfunction. Additionally, there is a high expression of genes involved in DNA repair, chromosomal instability, and proliferation. This subtype demonstrates the highest chemosensitivity, particularly to platinum-based agents. It has a higher pathological complete response (pCR) rate to neoadjuvant chemotherapy compared with subtype BL2. From the prognostic point of view, the prognosis is better if a pCR is achieved [13,14,15,16].

The BL2 subtype is associated with abnormalities in growth factor signaling, particularly in relation to the epidermal growth factor receptor (EGFR), nerve growth factor (NGF), insulin-like growth factor 1 receptor (IGF1R), mesenchymal–epithelial transition factor (MET), and the canonical Wnt/β-catenin pathway (Wnt) [12]. This subtype exhibits lower chemosensitivity compared to BL1, as well as lower pCR rates with standard chemotherapy. The prognosis of BL2 is poorer than that of BL1, particularly in patients with residual disease following neoadjuvant treatment [16,17].

The M subtype of TNBC is characterized by enrichment of genes associated with epithelial–mesenchymal transition (EMT), cell motility, and stemness. It confers on TNBC a more invasive and aggressive phenotype compared to the BL subtypes. The M subtype has features of EMT, cell differentiation, motility, migration, and invasion. It displays stem-cell–like features. It is associated with upregulation of the phosphatidylinositol 3-kinase/protein kinase B/mammalian target of rapamycin (PI3K/Akt/mTOR) signaling pathways. Additionally, from an immune profile perspective, the M subtype is generally considered an “immune-cold” cancer, with lower lymphocyte infiltration compared to the IM subtype. The main markers include mesenchymal markers [vimentin, N-cadherin, zinc finger E-box binding homeobox 1 (ZEB1), and twist-related protein 1 (TWIST1)] and stem cell markers [aldehyde dehydrogenase 1 (ALDH1) and CD44/CD24 ratio] [18].

Clinically, the M subtype of TNBC is characterized by a more invasive phenotype with high metastatic potential, including to the lungs and brain. It is less responsive to standard chemotherapy compared to the BL1 subtype, and is often associated with a poorer prognosis relative to the BL1 and IM subtypes [18,19].

A distinct molecular profile within the TNBC category typifies the IM subtype. Characterized by high expression of immune-related genes, this subtype reflects tumors with active immune signaling and increased numbers of tumor-infiltrating lymphocytes (TILs). Molecular features include enriched pathways, such as cytokine signaling (e.g., interferon-γ); antigen presentation; T-cell, B-cell, and natural killer (NK) cell signaling; and activation of immune checkpoint pathways, most prominently PD-1/PD-L1 and cytotoxic T-lymphocyte–associated protein 4 (CTLA-4). The main immune markers include a high density of CD8+ TILs and increased PD-L1 expression [20,21,22].

From a clinical perspective, the IM TNBC subtype has a better prognosis than either the M or BL subtypes of TNBC, provided it is appropriately treated. High numbers of TILs are associated with a better response to chemotherapy and immunotherapy and have been reported to correlate with pCR following neoadjuvant chemotherapy [20,21]. Notably, the IM subtype is considered “immune-hot”, making it a candidate for ICP-targeted therapy [20,21].

Patients with IM TNBC benefit from treatment with PD-1/PD-L1 inhibitors (e.g., pembrolizumab and other ICIs) in combination with chemotherapy [23,24]. Potential prognostic and/or predictive biomarkers include PD-L1 expression, TIL density, and an interferon-γ signature [20,21,22].

Subsets of TILs in TNBC include several subtypes of T-cells [CD4+, CD8+, T helper cells, and regulatory T-cells (Tregs)] and NK cells, which can be further categorized by their specific proteins (like CD56, CD57, granulysin, and granzyme B) and their spatial distribution within the tumor. High levels of specific TIL subsets, such as CD8+ T-cells and NK cells, are associated with better prognoses and favorable clinical outcomes. Notably, however, the spatial uniformity of TIL infiltration also impacts patient treatment outcomes [22,25,26].

LAR TNBC is a molecular subtype characterized by high expression of androgen receptor (AR)-related genes and hormone-regulated signaling pathways, despite being IHC-negative for ERs, PRs, and HER2. These tumors exhibit a luminal-like gene expression profile. Molecular markers include AR signaling, steroid hormone metabolism, and luminal cytokeratin (CK8/18) expression. Importantly, LAR TNBC is characterized by low proliferation compared to BL TNBC and low TIL density [27]. LAR tumors often harbor a high rate of activating mutations in *PIK3CA*, a component of the PI3K/Akt/mTOR signaling pathway. Indeed, *PIK3CA* is one of the most commonly mutated genes in TNBC (40%) and drives the growth of cancer cells, chemotherapy resistance, and the emergence of immunologically cold clones [28]. The LAR subtype of TNBC represents 10% to 15% of TNBC patients and is more common in older patients compared to the BL TNBC subtype. The clinical behavior of this TNBC subtype is typically less aggressive than that of BL or M TNBC. Notably, LAR TNBC is associated with low chemosensitivity and lower pCR rates after standard neoadjuvant chemotherapy [9,18,27].

Mutations involving *BRCA* are associated with TNBC, particularly *BRCA1* mutations, which increase the likelihood of developing TNBC [29]. The *BRCA1* gene mutations are associated with a more aggressive disease and an earlier age of onset. Although currently not standard of care, genetic testing for *BRCA* mutations should be performed routinely in TNBC, and should be an essential predictive biomarker, particularly for selecting patients for platinum-based chemotherapy and poly (ADP ribose) polymerase (PARP) inhibitors [29]; however, not all TNBC is linked to *BRCA* mutations, and not all *BRCA*-related BCs are TNBC [29].

Tumor mutational burden (TMB) measures the number of genetic mutations in a tumor. TNBC has a higher TMB compared to other BC subtypes, and a high TMB is considered an indicator of the potential to benefit from ICI therapies [30]. High TMB correlates with a greater number of neoantigens, which, in turn, stimulate the immune system to recognize and attack TNBC cells [30]. The molecular, clinical, and therapeutic differences between the BL1 and BL2 subtypes are summarized in Table 1 below.

## 4. Immune Checkpoint Proteins

Infiltration of T-cells into the tumor microenvironment (TME) is beneficial for favorable cancer outcomes [31]. In TNBC, T-cells can control tumor growth through immune editing [31]. The immune system distinguishes between self and non-self through the binding of T-cell receptors (TCRs) present on effector cytotoxic T-cells to peptides presented by major histocompatibility complex class 1 (MHC-1) molecules on the surfaces of all nucleated cells, including tumor cells. As T-cells need two signals for activation, the binding of the TCR with the MHC-1 molecule alone does not activate the T-cells. An interaction between co-inhibitory or co-stimulatory signals further impacts the TCR-MHC pathway. ICPs are molecules that can increase or decrease the signals of the immune system and can be divided into two groups: stimulatory molecules (e.g., CD80/CD28) and inhibitory molecules, such as PD-1, PD-L1, CTLA-4, and lymphocyte-activation gene 3 (LAG-3). Tumors upregulate these inhibitory signals to evade immune system attack [32,33,34]. The ICPs encompass ligand–receptor pairs that have a co-inhibitory or co-stimulatory effect on immune responses. Dendritic cells (DCs) orchestrate T-cell responses by presenting antigenic peptides on MHC-1. For the naïve T-cell to become an effector cell, a second transmembrane signal event is required. This second signal is provided by co-stimulatory or co-inhibitory molecules, which are expressed on antigen-presenting cells (APCs) and bind to the ligands of these molecules that are expressed on T-cells. Many signaling pathways diverge following ligand–receptor binding [35,36,37,38,39,40,41,42,43,44,45,46,47,48,49]; these are summarized in Table 2 below.

### Soluble Immune Checkpoint Proteins

ICPs are mostly membrane-bound receptors that are expressed by more than one type of immune cell population, and the expression of the ICPs varies within a particular cell type [50,51,52]. In addition to being membrane-bound, soluble forms of ICPs occur. Membrane-bound ICPs are located on cell membranes and exosomal membranes, while soluble ICPs are found in bodily fluids, such as plasma, serum, urine, cerebrospinal fluid, and peritoneal fluid [53]. Soluble ICPs bind to the same ligands as the membrane-bound forms of these proteins [53].

These soluble forms of ICPs are associated with systemic biological effects and often function as decoy receptors, binding to their ligands in the bodily fluids and preventing them from interacting with the membrane-bound receptors on the cell surface. These biological effects modulate cellular signaling and immune responses at a distance from the site of production. For example, a soluble inhibitory ICP can circulate and broadly suppress an immune response, rather than only affecting neighboring cells. Significantly, they may interfere with the therapeutic effect of the targeted monoclonal antibodies (mAbs) [54,55].

Measurement of systemic levels of soluble ICPs as diagnostic and prognostic biomarkers is a non-invasive and low-risk strategy and can be achieved by application of various quantitative immunoassays. An additional advantage of quantification of systemic soluble ICPs is that they can be measured over multiple time points during the course of disease and its treatment as an alternative to serial follow-up biopsies [53,56]. Importantly, serial measurement of the circulating levels of ICPs is potentially useful as a disease-monitoring strategy for ICI therapy [55].

Soluble ICPs are produced through the alternative splicing of messenger (m)RNA or the cleavage/shedding of membrane-bound ICPs [34,54,57]. Alternative splicing results in transcripts lacking the transmembrane region, which are secreted rather than remaining membrane-bound. Proteolytic shedding occurs when proteases [often matrix metalloproteinases and a disintegrin/ metalloproteinase (ADAM) family enzyme] cleave the membrane-bound ICPs at the extracellular domain. This cleavage releases the extracellular portion into circulation as the soluble form of the protein [55,58]. The mechanisms whereby the various soluble ICPs are generated, as well as their origins and biological activities, are summarized in Table 3 [34,42,44,53,55,56,57,58,59,60,61,62,63,64,65]. The functional significance of soluble ICPs is that they retain their immune-modulatory activity by binding to ligands or receptors, even in the absence of a cell membrane anchor. Accordingly, they are also involved in immune regulation [34]. It must be emphasized, however, that controversy exists in the literature regarding the significance of types and expression levels of soluble ICPs in various malignant diseases. To the best of our knowledge, there is no published comparison between the various immunoassays of soluble ICPs, particularly in clinical settings. Nevertheless, there is emerging interest in targeting these ICPs in the treatment of TNBC, as discussed in the section below.

## 5. Rationale for the Use of Immune Checkpoint Inhibitors in TNBC

Treatment of TNBC with ICIs involves combining these agents (pembrolizumab or atezolizumab) with chemotherapy to improve outcomes for PD-L1–positive metastatic TNBC [66,67]. In patients with early-stage TNBC, the use of ICIs (e.g., pembrolizumab) is recommended independent of PD-L1 expression [66,67]. Combination therapy has been shown to increase pCR rates compared to chemotherapy alone. Monotherapy with ICIs is not recommended due to their low efficacy in TNBC. Ongoing clinical trials are exploring new combinations with other targeted therapies, such as PARP inhibitors and antibody–drug conjugates, to enhance treatment effectiveness further [5].

The combination of ICI-targeted mAbs and chemotherapy augments the immune response by releasing tumor antigens/neoantigens. The ICIs block the PD-1/PD-L1 pathway, a common mechanism by which tumors evade the immune system. Anti–PD-L1 mAbs bind PD-L1 on tumor cells and APCs/DCs, preventing PD-L1 from engaging PD-1 on T-cells. This mechanism blocks the inhibitory signal, allowing exhausted or suppressed CD8+ T-cells to regain proliferation and cytokine production (such as IFN-γ) and restore their cytotoxic activity [68]. Importantly, the presence of TILs is a critical biomarker in TNBC [69]. The proportion of early TNBC patients with intermediate or higher levels (≥10%) of stromal TILs is approximately 50% [69,70]. Additionally, TILs are a strong prognostic and predictive biomarker of ICP blockade in early TNBC [21,71].

PD-L1 blockade increases the infiltration and activity of effector T-cells and can reduce suppressive cell populations, such as Tregs and myeloid-derived suppressor cells (MDSCs). Additionally, this therapeutic strategy promotes an inflammatory (“hot”) TME, enabling effector cells to act efficiently. These changes also enhance antigen presentation and crosstalk with innate effectors, such as macrophages and NK cells [72].

Additional evidence for combining cytotoxic chemotherapy with ICIs is that chemotherapy can increase tumor antigen release, leading to immunogenic cell death, upregulation of MHC-1 and PD-L1 expression, and depletion of immunosuppressive populations (Tregs and MDSCs) [73,74]. PD-L1 expression on immune cells or tumor cells and high numbers of TILs correlate with a higher likelihood of benefit. TNBC is more immunogenic than other forms of BC due to its higher TMB (10 mutations per megabase), with an intermediate-to-high proportion of patients exhibiting significant infiltration of TILs and high expression of PD-L1, providing an additional rationale for incorporating anti–PD-1 and anti–PD-L1 ICIs in the treatment of TNBC [66,75,76].

### 5.1. Incorporation of Pembrolizumab and Atezolizumab in the Treatment of TNBC

Pembrolizumab was approved by the Food and Drug Administration (FDA) in combination with chemotherapy as the first-line treatment for patients with locally recurrent, unresectable, or metastatic TNBC in 2020. The indication was given to patients with PD-L1–positive tumors [combined positive score (CPS) ≥ 10 on the Dako 22C3 assay] and a disease-free interval ≥ 6 months. The approval was based on the results of a Phase III KEYNOTE-355 trial (multicenter, double-blinded, randomized, placebo-controlled study). The study included 566 patients who received pembrolizumab plus chemotherapy and 281 who received placebo plus chemotherapy. Backbone chemotherapy included carboplatin combined with paclitaxel, nab-paclitaxel, or gemcitabine. The effect was clearly shown in patients with a CPS ≥ 10. A median progression-free survival of 9.7 months was observed in the pembrolizumab–chemotherapy arm relative to 5.6 months in the chemotherapy–placebo arm [hazard ratio (HR): 0.65; 95% CI: 0.49–0.86; *p* = 0.0012]. Furthermore, there was a significant improvement in overall survival with respective durations of 23.0 months compared to 16.1 months (HR: 0.73; 95% CI: 0.55–0.95; *p* = 0.0185) [77].

KEYNOTE-522 was a Phase III clinical trial that investigated the use of pembrolizumab combined with neoadjuvant chemotherapy for high-risk, early-stage TNBC. Backbone chemotherapy included four cycles of paclitaxel with carboplatin and pembrolizumab, followed by four cycles of doxorubicin (or epirubicin) with cyclophosphamide (AC/EC) and pembrolizumab. Following surgery, patients received adjuvant pembrolizumab (i.e., pembrolizumab alone) for up to nine cycles (unless recurrence or unacceptable toxicity) in the pembrolizumab arm (or placebo in the control arm). The primary endpoints were pCR and event-free survival. The study showed that this regimen significantly improves pCR rates [78]. In earlier reports, pCR rates were significantly higher with pembrolizumab and chemotherapy (64.8%; 95% CI: 59.5–66.4%) than with placebo and chemotherapy (55.6%; 95% CI: 50.6–60.6%). The estimated treatment difference was approximately 7.5% (95% CI: 1.6–13.4%) in favor of the pembrolizumab regimen; this difference was statistically significant (*p*-value = 0.00055). At 36 months, the event-free survival was 84.5% in the pembrolizumab arm compared to 76.8% in the placebo arm (HR: 0.63, favoring pembrolizumab) [23]. With longer follow-up, the 5-year (60-month) overall survival was 86.6% (95% CI: 84.0 to 88.8) in the pembrolizumab and chemotherapy arm vs. 81.7% (95% CI: 77.5 to 85.2) in the placebo and chemotherapy arm (*p* = 0.002) [78].

Atezolizumab is an anti–PD-L1 antibody originally approved for metastatic TNBC, in combination with nab-paclitaxel for PD-L1–positive tumors (*VENTANA^®^ PD-L1 (SP263) Assay:* 142 ≥1% immune cells). This agent was initially approved based on the results of the IMpassion130 trial; however, the FDA withdrew approval in 2021 due to subsequent trial results showing limited benefit. Nevertheless, this regimen is sometimes used in other geographic regions [79].

Although the introduction and incorporation of immune checkpoint blockade constitutes a significant improvement in the treatment of TNBC patients, the results are far from optimal. Clearly, there is an unmet medical need for identification of additional targets, such as the BTLA/HVEM/CD160 axis, as described below.

### 5.2. Mechanisms of Resistance to Immune Checkpoint Inhibitors in TNBC

**Figure 1:**
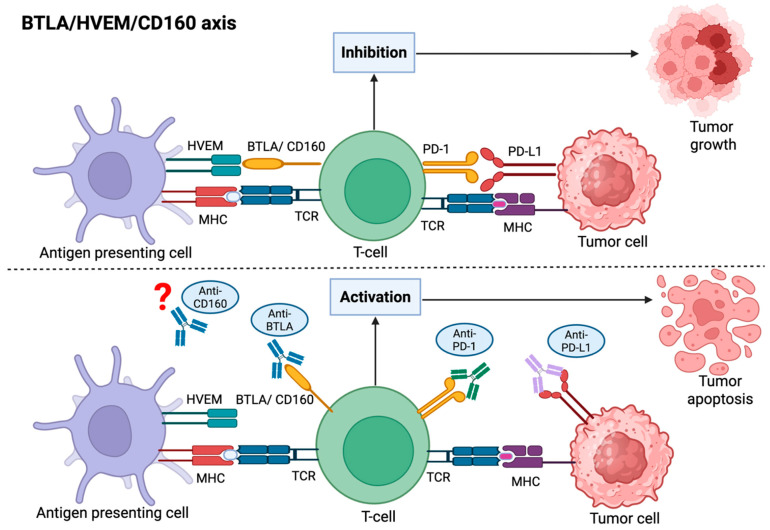
BTLA/HVEM/CD160 axis as potential therapeutic targets in triple-negative breast cancer. Created in BioRender. Rossouw, T. (2026) https://BioRender.com/v4df6t9, accessed on 27 April 2026.

Resistance mechanisms to ICIs include irreversible T-cell exhaustion and dysfunction, low or absent neoantigen burden, and defects in antigen presentation due to decreased expression of beta-2 microglobulin (β2M)/MHC-1 [22,80]. Importantly, upregulation of alternative inhibitory pathways, such as the BTLA/HVEM axis, and the presence of a highly suppressive TME represent additional operational mechanisms of resistance [81,82]. Blockading of the BTLA/HVEM axis may, therefore, confer additional anti-tumor activity [82]. A recent Phase I study evaluated HFB200603 (a BTLA-antagonist mAb) as monotherapy and in combination with the anti–PD-1 mAb tislelizumab in adult patients with advanced solid tumors [83]. This study demonstrated that HFB200603 has favorable safety and was associated with peripheral T-cell activation. A second study evaluated tifcemalimab (a recombinant, humanized IgG4k mAb targeting BTLA) in combination with toripalimab (a PD-1 inhibitor) in previously treated advanced lung cancer patients [84]. This combination showed promising anti-tumor activities with acceptable safety, especially in advanced refractory small-cell lung cancer. As depicted in Figure 1, future studies should evaluate the combination of anti–BTLA and anti–PD-1/PD-L1 in patients with TNBC in the metastatic setting and, possibly, in the neoadjuvant setting. Other possible combinations include anti–PD-1/PD-L1 with an anti-CD160 mAb. The activities of the components of the BTLA/HVEM/CD160 axis are covered in greater detail in Section 6.

Antigen-presenting cells (APCs) and tumor cells present antigens to the T-cell receptor (TCR) via the major histocompatibility complex (MHC). The binding of programmed cell death protein 1 (PD-1) on T-cells with its ligand PD-L1 on tumor cells inhibits T-cell activity. Similarly, the binding of herpes virus entry mediator (HVEM) on APCs with the B- and T-lymphocyte attenuator (BTLA) leads to inactivation of T-cells and, hence, promotes tumor growth. Blocking of PD-1 or PD-L1 by monoclonal antibodies (mAbs) prevents their binding, allowing exhausted or suppressed T-cells to regain proliferation and cytotoxic capacity. The administration of an anti–BTLA mAb similarly inhibits the binding of BTLA on T-cells to HVEM on APCs or tumor cells, thereby activating T-cells and ultimately inducing tumor apoptosis. It is possible that a mAb targeting CD160 might have a similar effect, but, due to its complex functional role, more research is needed.

## 6. The B- and T-Lymphocyte Attenuator/Herpes Virus Entry Mediator/CD160 Pathway

The BTLA/HVEM axis has been convincingly demonstrated by transcriptomic expression of 397 genes in various solid tumors from 514 patients covering 32 different tumor types [81].

### 6.1. B- and T-Lymphocyte Attenuator

Soluble BTLA originates primarily from alternative RNA splicing of the BTLA gene, as well as from the shedding of the membrane-bound BTLA protein [54]. Soluble BTLA can also act as a decoy receptor, binding to its ligands and thereby inhibiting their interaction with the membrane-bound BTLA on cell surfaces. Additionally, by interfering with the BTLA/HVEM axis, soluble BTLA may modulate immune responses. Furthermore, soluble BTLA can inhibit the function of activating receptors on NK and T-cells, contributing to immune suppression in patients with chronic lymphocytic leukemia [85].

Mechanistically, unlike PD-1 and CTLA-4, which contain a variable-type immunoglobulin (IgV) extracellular domain, BTLA has a single intermediate-type immunoglobulin (IgI) domain. The cytoplasmic tail contains two immunoreceptor tyrosine-based inhibitory motifs (ITIMs) and a growth factor receptor-bound protein 2 (Grb2)-recognition consensus site. Notably, HVEM serves as the only ligand for this ICP, providing a predominantly inhibitory signal through the ITIMs and recruitment of Src homology region 2 domain-containing protein tyrosine phosphatase (SHP)-1 and SHP-2, which limit stimulatory pathways activated by tyrosine kinases [85,86]. BTLA has also been shown to provide stimulatory signaling in TILs used in adoptive T-cell therapy through interactions of Grb2-linked Src homology 2 domain-containing leukocyte protein of 76 kDa (SLP-76) and the oncogene, Vav [87].

### 6.2. Herpes Virus Entry Mediator

HVEM is expressed on various immune cells, including T-cells, Tregs, B-cells, NK cells, monocytes, and DCs. It is also found on non-immune cells, such as epithelial and mesenchymal cells [88]. Soluble isoforms of HVEM derive primarily from the shedding of the membrane-bound HVEM protein from cell surfaces or through a mechanism of alternative mRNA splicing [54]. These isoforms function as immune checkpoint regulators, modulating immune responses by engaging with their ligands.

Soluble HVEM can act as a decoy receptor, binding to its ligands BTLA, CD160, and LIGHT (lymphotoxin-like, exhibits inducible expression, and competes with HSV glycoprotein D for HVEM, a receptor expressed by T lymphocytes), thereby inhibiting their interaction with membrane-bound HVEM on cell surfaces, potentially resulting in attenuation of the immune response. Notably, this biological effect differs from membrane-bound HVEM, which serves as a receptor on the surface of immune cells, receiving signals from various ligands to activate or suppress immune responses [89]. In some malignancies, such as acute lymphocytic leukemia and experimental models of prostate cancer, HVEM is associated with tumor immune escape by inhibiting T-cell function [90,91]. Notably, HVEM may be a potential biomarker in numerous cancers and other non-malignant diseases [86,89,92,93].

Mechanistically, the extracellular domain consists of four cysteine-rich domains (CRD1–4), which create two distinct binding regions for various ligands. The CRD2 and CRD3 domains of the LIGHT [also known as the tumor necrosis factor receptor superfamily (TNFSF)14] binding region bind LIGHT and lymphotoxin-α (LT-α). CRD1 forms the Duffy antigen receptor for chemokines (DARC) binding region, which binds BTLA, CD160, and the herpes simplex virus glycoprotein D (HSV gD) [86].

The short cytoplasmic tail of the membrane-bound HVEM contains a binding site for the TNFR-associated factors (TRAFs) family of ubiquitin E3 ligases, specifically TRAF2, which mediates downstream nuclear factor kappa-light-chain enhancer of activated B-cell (NF-ĸB) signaling to promote inflammation and cell survival [85,86]. Importantly, binding of HVEM with either BTLA or CD160 results in an inhibitory signal in T-cells. In contrast, when binding to either LIGHT or LT-α, T-cells are activated [94]. In addition to activating its own intracellular signal transduction pathways, HVEM may also serve as a ligand depending on the corresponding receptor. Furthermore, membrane-bound HVEM signaling behavior is influenced by the spatial relationship to its binding partner, BTLA. HVEM binding BTLA in the *trans* complex enhances NF-ĸB signaling, whereas HVEM binding BTLA in the *cis* complex suppresses NF-ĸB signaling by preventing HVEM from binding to BTLA and CD160 in the *trans* configuration [85,86].

### 6.3. CD160

CD160 is a type V transmembrane protein and a member of the immunoglobulin superfamily. CD160 has two different cell surface anchors, a glycosylphosphatidylinositol (GPI) anchor and a transmembrane form, which are generated through alternate RNA splicing. A soluble form of CD160 is generated through the cleavage of CD160-GPI by phospholipases [85,86]. Expression of CD160 is mostly limited to T-cells, NK cells, and NKT cells and shows upregulation on activated CD8+ and CD4+ T-cells [86]. NK cells constitutively express the CD160-GPI isoform and upregulate CD160 transmembrane (CD160-TM) expression upon activation. CD160 has been reported to show aberrant expression in some B-cell malignancies, such as chronic lymphocytic leukemia [86]. HVEM, as well as classical and non-classical MHC-1 molecules, have been identified as ligands for CD160. Interestingly, despite both membrane-bound isoforms having an identical extracellular domain, the interactions of CD160-TM are limited to only MHC-1 molecules [95].

The functional role of CD160 is complex and dependent on the isoform, expressing cell, interacting ligand, and immune context. CD160-GPI binding to HVEM forms a bidirectional signaling pathway. CD160-GPI engagement to HVEM (expressed on APCs) induces stimulatory signaling in the HVEM-expressing cell, whereas the CD160-GPI-expressing cell receives predominantly inhibitory signaling depending on the immune context [86,94,95]. In NK cells, cytolytic activity is blocked by soluble CD160, whereas the membrane-bound forms increase NK-cell cytolytic activity through recruitment of Src-family kinase p56 (Lck) and activation of the extracellular signal-regulated kinase 1 and 2 (Erk1/2) pathway [85,86].

### 6.4. Additional Stimulatory Ligands

Other ligands of HVEM include LIGHT and LT-α, and these are described below.

#### 6.4.1. Lymphotoxin-like, Exhibits Inducible Expression, and Competes with HSV Glycoprotein D for HVEM

Lymphotoxin-like, exhibits inducible expression, and competes with HSV glycoprotein D for HVEM, a receptor expressed by T lymphocytes (LIGHT), is a type II transmembrane protein and forms part of the TNFRS [85,86]. Expression of LIGHT is inducible on immature DCs, T-cells, B-cells, and NK cells. It is also found in both membrane-bound and soluble forms. Soluble LIGHT is formed through selective proteolytic cleavage of the ectodomain by metalloproteinases [86]. Both membrane-bound and soluble LIGHT can serve as ligands for HVEM with subsequent activation of NF-ĸB signaling, resulting in a significant pro-inflammatory response, potentially increasing the anti-tumor response [96]. Furthermore, membrane-bound LIGHT may cause dissociation of HVEM from BTLA in the *trans* form due to its higher binding affinity for HVEM [85,86]. The expression and bioavailability of LIGHT are regulated by a variety of mechanisms, including modulation of transcription, as well as by elevated serum levels of soluble decoy receptor-3 (DcR3), which, through its binding with LIGHT, reduces the bioavailability of the protein [85,86].

#### 6.4.2. Lymphotoxin-α

Lymphotoxin-α, otherwise known as tumor necrosis factor β (TNF-β), is a member of the TNFRS. LT-α is produced by a variety of immune cells, including macrophages, T-cells, B-cells, and NK cells. It is secreted in a soluble form but may form heterotrimeric complexes with lymphotoxin-β (LT-β) on the cell surface. Assembly of LT-α into trimers is required to form a receptor-binding site that enables binding with its receptors, unlike the homotrimeric and heterotrimeric forms (with LT-β), which exhibit a low binding affinity for HVEM [85,86,94].

## 7. The Role of the BTLA/HVEM/CD160 Pathway in Breast Cancer and TNBC

Given the paucity of published research on the involvement of the BTLA/HVEM axis in both the pathogenesis and treatment of TNBC and its various subtypes (BL1, BL2, IM, and LAR), we have included the role of this pathway in breast cancer in general. The BTLA/HVEM pathway has been shown to play a significant role in cancer immunopathogenesis. HVEM expression is seen in many solid cancers and serves as a potential prognostic biomarker, correlating with more advanced disease and poor patient outcomes due to its predominantly immunoinhibitory interactions. Expression of HVEM is associated with decreased TILs, decreased cytotoxic cell activity, and decreased effector cytokine production, such as IFN-γ [97]. BTLA also plays a predominantly oncogenic role. Increased BTLA expression, including its soluble form, indicates an unfavorable prognosis in multiple solid tumors (lung cancer, melanoma, gastrointestinal malignancies, oral malignancies, and gynecological malignancies), which is related to increased metastasis and decreased overall survival [64,98,99]. A negative correlation is seen between BTLA expression and the proportion of infiltrating cytotoxic CD3+ CD8+ T-cells in patients with solid tumors, indicating T-cell suppression as a result of increased BTLA expression by tumor cells [98]. Furthermore, upregulation of BTLA on NK cells also inhibits their anti-tumor response due to reduced cytotoxicity and IFN-γ production [98]. This is supported by improved anti-tumor activity in murine models of experimental immunotherapy following targeted attenuation of BTLA expression by NKT type 1 cells [100]. BTLA may also play a role in resistance to currently available immunotherapy (PD-1 inhibition) in BC. It has been demonstrated in isolated human cytotoxic T-cells from BC patients that PD-1 expression does not necessarily equate to complete attenuation of immune reactivity [101]. This emphasizes the role of other ICPs, such as BTLA, in T-cell deactivation in BC, underscoring the potential role of co-targeting ICPs in the management of BC. BTLA and PD-1 have structural similarities, with both containing an IgV domain in the extracellular region and both containing ITIMs and immunoreceptor tyrosine-based switch motifs (ITSMs) in their cytoplasmic tails. In this context, PD-1 has a far greater preference for recruiting SHP-2 over SHP-1 (29-fold), whereas BTLA, while preferentially recruiting SHP-1, uses SHP-1 and SHP-2 in a more balanced way. PD-1, therefore, blocks phosphorylation of CD28 via SHP-2 recruitment, while BTLA blocks phosphorylation of both CD28 and the CD3ζ subunit of the TCR via SHP-2 and SHP-1 recruitment, respectively. TCR inhibition by SHP-1 inhibits interleukin-2 production more effectively compared to CD28 inhibition by SHP-2 [98]. Dual blockade of BTLA and PD-1 may, therefore, potentially act synergistically and improve immunotherapy outcomes in cancer [102]. In this context, experimental models have shown that combining anti–BTLA and anti–PD-1 mAbs augments both CD4+ and CD8+ T-cell proliferation. This contrasts with combinations of PD-1–targeted mAbs with other ICIs that only augment the proliferation of one T-cell subset. Additionally, T-cells activated in the setting of a dual BTLA and PD-1 blockade promoted potent cytotoxic capacity [103]. Further evidence of the dual blockade was shown in murine models of glioblastoma, with improved long-term overall survival seen in the dual anti–BTLA/anti–PD-1 arm (60%) compared to the individual anti–PD-1 (20%) and anti–BTLA (0%) arms. The effect was consistent with increased CD4+ and CD8+ T-cell activity and decreased Treg-cell activity [102].

With respect to immunopathogenesis, HVEM is predominantly pro-oncogenic through direct stimulation of cell cycle progression in HVEM-expressing tumor cells and indirectly through its interaction with its inhibitory receptor (BTLA) on T-cells and other immune cells. Membrane expression of HVEM (mHVEM) in BC was found in different molecular subtypes. The highest expression was seen in HER2-overexpressing BC, followed by TNBC, with 66.7% and 24.6% of cases involved, respectively. HVEM-positive BC was significantly associated with more aggressive tumor phenotypes, including higher tumor grade, larger size, and higher nodal stage. HVEM-positive BC with low TILs showed the worst disease-free and overall survival [103]. Additionally, the serum levels of soluble HVEM in BC were also shown to be significantly elevated in stages III and IV compared to earlier stages [104]. Two studies evaluating the pre-treatment levels of ICPs reported significantly lower plasma levels of HVEM and a numerically non-significantly lower plasma level of BTLA in early BC (predominantly stages I and II) [105,106]. These observations require further investigation and support the use of the measurement of soluble HVEM as a potential prognostic biomarker in BC.

Increased levels of BTLA RNA have been described in patients with BC. The high RNA expression of BTLA was also associated with increased RNA expression of other ICPs, including CTLA-4, PD-1, HVEM, and CD160 [81]. In luminal BC, higher BTLA RNA expression was reported in metastatic compared to non-metastatic disease [107].

The role of CD160 in the immunopathogenesis of BC has recently been investigated. Using machine learning methods, *CD160* was identified as one of seven key genes in a TNBC immunophenotypic signature with prognostic implications [108]. Alterations in peripheral-blood *CD160* cell-free DNA methylation patterns have also been associated with BC; however, these differ with respect to various patient populations, indicative of ethnic differences. In this context, hypomethylation of *CD160* was reported in BC patients of Chinese descent, in contrast to hypermethylation of *CD160* seen in a cohort of women of European descent [109,110]; this observation requires further investigation.

Regarding the use of CD160, HVEM, and BTLA as potential indicators for patient selection, it is important to point out that, for clinical use, these biomarkers require further validation, as the specificity and sensitivity have not been standardized.

Within the TME, immunosuppressive components such as myeloid-derived suppressor cells, cancer-associated fibroblasts (CAFs), as well as extracellular matrix remodeling further reinforce checkpoint signaling through cytokine production, stromal interactions, and physical barriers to immune infiltration [111]. Interaction of the BTLA/HVEM/CD160 axis with these stromal and myeloid components of the TME likely contributes further to T-cell dysfunction and tumor progression [112]. In melanoma, CAFs have been shown to exhibit elevated expression of HVEM. Exposure of cytotoxic T-lymphocytes to CAF-conditioned media leads to increased expression of BTLA, suggesting a mechanism by which interactions between T-cells and melanoma-associated fibroblasts promote T-cell exhaustion [113].

In the case of TNBC, immunophenotypic assessment by IHC and flow cytometry revealed increased CD160 expression. Interestingly, only the CD160-TM isoform was expressed on the TNBC cells. CD160 may, therefore, serve as a potential biomarker and therapeutic target for this subtype of BC. In this context, a study by Scheffges et al.—based on administration of an anti-CD160-TM mAb (22B12) with agonistic NK cell cytotoxic activity—showed promise as a future therapeutic agent in TNBC patients. In addition, pre-clinical in vivo investigations based on administration of CD160-TM-targeted mAbs (22B12) in TNBC murine models have demonstrated anti-tumor efficacy mediated via enhanced NK cell cytotoxicity and phagocyte-mediated killing of TNBC cells [95].

## 8. Conclusions

The treatment of patients with TNBC remains highly challenging. Despite remarkable advances in cancer immunotherapy, outcomes for many patients remain poor, and a significant unmet need persists. A substantial proportion of TNBC patients continue to experience disease progression, even with current therapeutic options, and a high proportion will ultimately succumb to the disease. These limitations highlight the urgent need for more effective, durable, and accessible treatment strategies that can meaningfully improve survival and quality of life for this patient population. Future studies should validate targeting the BTLA/HVEM/CD160 axis for the treatment of TNBC, and importantly, to stratify these biomarkers for the various subtypes.

## Figures and Tables

**Table 1:** Molecular, clinical, and therapeutic differences between the BL1 and BL2 subtypes of TNBC.

Feature	BL1	BL2	Refs.
Dominant pathways	Cell cycle, DNA damage response	Growth factor signaling (EGFR, NGF, IGF1R, MET, Wnt)	[12,13,14,16]
Proliferation	Very high (52%)	Moderate (0–~23%)	[14]
*BRCA1* link	Strong	Less frequent	[16]
Chemotherapeutic response (especially to platinum)	High (best among TNBC subtypes) (34%)	Lower (20%)	[14,15]
pCR rate	High (52%)	Low (0–~23%)	[16,17]
Prognosis	Better (OS = 45 months)	Poorer (OS = 19 months)	[17]

Abbreviations: BL: basal-like; *BRCA1*: breast cancer gene 1; DNA: deoxyribonucleic acid; EGFR: epidermal growth factor receptor; IGF1R: insulin-like growth factor 1 receptor; MET: mesenchymal epithelial transition factor; NGF: nerve growth factor; OS: overall survival; pCR: pathological complete response; TNBC: triple-negative breast cancer; Wnt: Wnt/β-catenin pathway.

**Table 2:** The major pathways that diverge downstream following immune checkpoint protein/ligand interaction.

ICP Receptor	ICP Ligand	Key Phosphatase/ Kinase	Primary Pathway Inhibited	Refs.
PD-1	PD-L1/PD-L2	SHP-2	PI3K/Akt/mTOR, RAS/MEK/ERK, TCR proximal signaling	[35,36,37,38,39]
CTLA-4	CD80/CD86	PP2A, SHP-2	PI3K/Akt/mTOR, TCR-CD3 complex activation	[38,39,40,41]
LAG-3	MHC-II/FGL-1/ GAL-3/LSECtin	“KIEELE” and “EP” motif	TCR–CD3 complex, Ca^2+^ flux, NFAT/NF-ĸB	[38,39,42,43,44,45]
TIM-3	GAL-9/ PtdSer/ HMGB-1/CEACAM-1	Fyn recruitment via Bat3 release	TCR signaling, NFAT activation	[38,39,46,47]
BTLA	HVEM	SHP-1/SHP-2	PI3K/Akt/mTOR, NF-ĸB/MAPK, TCR signaling	[48,49]

Abbreviations: Bat3: human leukocyte antigen-B–associated transcript 3; Ca^2+^: calcium; EP: glutamic acid–proline-rich tandem repeat; Fyn: Src family tyrosine kinase; CEACAM: carcinembryonic antigen-related cell adhesion molecules; FGL-1: fibrinogen-like protein-1; GAL: galectin; HMGB-1: high mobility group box protein 1; KIEELE: amino acid sequence (lysine/isoleucine/glutamic acid/glutamic acid/leucine/glutamic acid); LSECtin: liver and lymph node sinusoidal endothelial cell C-type lectin; MAPK: mitogen-activated protein kinase; NFAT: nuclear factor of activated T-cells; MHC-II: major histocompatibility complex Class II; NF-ĸB: nuclear factor kappa-light-chain enhancer of activated B-cells; PI3K/Akt/mTOR: phosphatidylinositol 3-kinase/protein kinase B/mammalian target of rapamycin; PtdSer: phosphatidylserine; SHP: Src homology region 2 domain-containing protein tyrosine phosphatase; TCR: T-cell receptor.

**Table 3:** Mechanisms whereby key inhibitory soluble immune checkpoint proteins are generated and their primary effect on T-cells.

Soluble ICP	Origin of ICP	Mechanism	T-Cell Response	Refs.
sPD-1	Activated T-cells, B-cells, NK cells, Tregs, monocytes, DCs, macrophages	Alternative mRNA splicing	T-cell exhaustion, reduced proliferation, reduced cytokine production (IL-2 and IFN-γ)	[34,53,55,59,60]
sPD-L1	APCs (B-cells, macrophages, DCs), non-classical APCs (tumor cells), mast cells, vascular endothelial cells, tumor cells	Alternative mRNA splicing/protein cleavage	Attenuates anti-tumor activity of T-cells	[53,55,61]
sCTLA-4	Activated T-cells, B-cells, NK cells, NKT cells, DCs, Tregs, tumor cells	Alternative mRNA splicing	Inhibits interaction of CD28 on T-cells with CD80/86 on APCs, attenuates T-cell differentiation, inhibits IL-2 production	[34,53,60,62]
sTIM-3	Activated T-cells, monocytes/macrophages, DCs, Tregs	Alternative mRNA splicing/protein cleavage	Attenuates T-cell-driven immune responses	[53,60,63]
sLAG-3	CD4+/CD8+ T-cells, NK cells, DCs	Protein cleavage	T-cell exhaustion, reduced cytokine production (IL-2 and IFN-γ), attenuates anti-tumor activity of T-cells	[42,44,53,60]
sBTLA	CD4+/CD8+ T-cells, B-cells, DCs, macrophages, monocytes, NK cells, NKT cells	Alternative mRNA splicing/cleavage of membrane-bound protein	Attenuated T-cell proliferation, reduced cytokine production (IL-10 and IFN-γ), induces peripheral tolerance of CD4+/CD8+ T-cells	[53,55,64]
sHVEM	T-cells, B-cells, NK cells, Tregs, DCs, monocytes	Protein cleavage	Promotes T-cell activation and proliferation (bound to LIGHT), dampens T-cell response (bound to BTLA or CD160)	[53,65]

Abbreviations: APCs: antigen-presenting cells; BTLA: B- and T-lymphocyte attenuator; CD: cluster of differentiation; CTLA-4: cytotoxic T-lymphocyte–associated protein 4; DCs: dendritic cells; HVEM: herpes virus entry mediator; IL: interleukin; IFN-γ: interferon gamma; LAG-3: lymphocyte-activation gene 3; LIGHT: lymphotoxin-like, exhibits inducible expression, and competes with HSV glycoprotein D for HVEM, a receptor expressed by T lymphocytes; mRNA: messenger ribonucleic acid; NK: natural killer; PD-1: programmed cell death protein 1; PD-L1: programmed death-ligand 1; s: soluble; TIM-3: T-cell immunoglobulin and mucin-domain-containing-3; Tregs: regulatory T-cells.

## Data Availability

No new data were created or analyzed in this study. Data sharing is not applicable to this article.
